# Xanthine Oxidoreductase Inhibitor Use Associated With Reduced Risk of Sarcopenia and Severe Sarcopenia in Patients Undergoing Hemodialysis

**DOI:** 10.3389/fmed.2022.817578

**Published:** 2022-02-07

**Authors:** Masafumi Kurajoh, Katsuhito Mori, Mizuki Miyabe, Shota Matsufuji, Akane Kizu, Yoshihiro Tsujimoto, Masanori Emoto

**Affiliations:** ^1^Department of Metabolism, Endocrinology, and Molecular Medicine, Osaka City University Graduate School of Medicine, Osaka, Japan; ^2^Department of Nephrology, Osaka City University Graduate School of Medicine, Osaka, Japan; ^3^Division of Internal Medicine, Dialysis Center, Inoue Hospital, Osaka, Japan; ^4^Division of Rehabilitation, Inoue Hospital, Osaka, Japan

**Keywords:** XOR inhibitor, hemodialysis, sarcopenia, prevalence, AWGS 2019

## Abstract

**Background:**

Xanthine oxidoreductase (XOR) inhibition reduces reactive oxygen species (ROS) production and enhances adenosine triphosphate (ATP) synthesis. We investigated the protective effects of XOR inhibitor treatment on sarcopenia, frequently observed in patients undergoing hemodialysis (HD), in which increased ROS and ATP shortage are known to be involved.

**Methods:**

This retrospective cross-sectional study included 296 HD patient (203 males, 93 females). Muscle mass, physical performance, and muscle strength were assessed using dual-energy X-ray absorptiometry, five-time chair stand testing, and handgrip strength, respectively. The Asian Working Group for Sarcopenia 2019 criteria were used to define low muscle mass, low physical performance, and low muscle strength, as well as sarcopenia and severe sarcopenia.

**Results:**

Sarcopenia and severe sarcopenia prevalence rates were 42.2 and 20.9%, respectively. XOR inhibitor users (*n* = 119) showed a significantly (*p* < 0.05) lower prevalence of sarcopenia and severe sarcopenia, as well as reduced muscle mass, physical performance, and muscle strength than non-users (*n* = 177). Multivariate logistic regression analyses also revealed XOR inhibitor use to be significantly associated with low muscle mass [odds ratio (OR), 0.384; 95% confidence interval (CI), 0.183–0.806; *p* = 0.011] and low physical performance (OR, 0.286; 95% CI, 0.142–0.578; *p* < 0.001), while significance with low muscle strength was borderline. Furthermore, XOR inhibitor use was significantly associated with sarcopenia (OR, 0.462; 95% CI, 0.226–0.947; *p* = 0.035) and severe sarcopenia (OR, 0.236; 95% CI, 0.091–0.614; *p* = 0.003).

**Conclusions:**

XOR inhibitor use was significantly associated with reduced risk of sarcopenia/severe sarcopenia in HD patients, suggesting that XOR inhibitor treatment has protective effects on sarcopenia in HD patients.

## Introduction

Sarcopenia is characterized as a decline in skeletal muscle mass and function (1), and frequently observed in patients undergoing hemodialysis (HD) (2, 3), which is termed uremic sarcopenia. Furthermore, HD patients have increased mortality (4–6), which has been shown to occur a higher rate in those with as compared to without sarcopenia (3, 7), thus emphasizing the need for prevention and treatment of this conditions in patients undergoing that therapy.

Xanthine oxidoreductase (XOR) is an enzyme that generates reactive oxygen species (ROS), and also catalyzes oxidation from hypoxanthine to xanthine and then xanthine to uric acid in the purine degradation pathway (8). Thus, inhibition of XOR reduces ROS production, while it also enhances adenosine triphosphate (ATP) synthesis along with an increase in the purine salvage pathway by use of hypoxanthine (9–12). Since increased ROS and ATP shortage in skeletal muscle are known to be involved in sarcopenia, especially uremic sarcopenia cases (13–15), we speculated that XOR inhibitor treatment has effects to protect against sarcopenia through preservation of skeletal muscle mass and function by decreased ROS production and ATP enhancement in HD patient skeletal muscle tissues.

However, to the best of our knowledge, no report showing the effects of XOR inhibitor treatment on sarcopenia has been presented. To evaluate possible protective effects on sarcopenia, the present study was conducted to examine the association of XOR inhibitor use with sarcopenia in HD patients based on the Asian Working Group for Sarcopenia (AWGS) 2019 criteria (16).

## Materials and Methods

### Study Design

This was a retrospective cross-sectional observational study conducted at Inoue Hospital (Osaka, Japan), where HD treatment was generally performed three times per week for about 4 h each time. As part of routine clinical care at that institution, muscle mass, physical performance, and muscle strength in HD patients are generally measured at the start of the first HD session that occurs during the week of their birthday. The association of XOR inhibitor use with sarcopenia and its components was investigated.

### Study Participants

The study participants were enrolled based on the following criteria. Inclusion criteria: (1) patient undergoing HD, and (2) measurements of muscle mass, physical performance, and muscle strength performed between January 2018 and December 2018, and (3) age ≥20 years. Exclusion criteria: (1) HD therapy period <6 months, (2) presence of debilitating disease, such as liver cirrhosis, malignancy, infection, or acute illness, and (3) treated with corticosteroids.

The need/requirement for informed consent was waived by The Inoue Hospital Ethics Committee (approval no. 236) owing to the retrospective nature of the investigation. Following approval of the study protocol, all data subjected to analysis were collected from relevant patient medical records. This study was conducted in full accordance with the Declaration of Helsinki.

### XOR Inhibitor User

An XOR inhibitor user was defined as a patient treated with allopurinol, febuxostat, or topiroxostat, each of which have been approved for therapeutic use as an XOR inhibitor in Japan.

### Determination of Muscle Mass

Muscle mass was assessed using whole body dual-energy X-ray absorptiometry (Horizon A; Hologic, Massachusetts, USA) (17). Extremity fat-free mass minus bone mineral contents was considered to be appendicular skeletal muscle, and height-adjusted muscle mass was calculated as appendicular skeletal muscle divided by height in meters squared (kg/m^2^) (3). Low muscle mass was defined as height-adjusted muscle mass <7.0 kg/m^2^ for males and <5.4 kg/m^2^ for females (16).

### Measurement of Physical Performance

Physical performance was assessed using a five-time chair stand test. Patients were asked to stand and sit as fast as possible five times from a sitting position on a chair with a seat 40 cm high and straight back without arm rests, with their arms crossed on the chest and hands on their shoulders, as previously reported in the CHAIR [change hemodialysis patients' activity and impaired functions by chair stand exercise] study (18). They initiated performance of the test when an experienced research staff member spoke the word “start” and the time (seconds) until the end of the fifth repetition was recorded (19, 20). Low physical performance was defined as a test time ≥12 s (16).

### Measurement of Muscle Strength

Muscle strength was assessed by determining handgrip strength using a Smedley type hand dynamometer (T.K.K.5101; Takei, Niigata, Japan), performed by an experienced research staff member (21, 22). Patients were instructed to hold the grip in an upright position with maximum force and with their arm extended. Measurements were repeated two times with each hand and the highest value (kg) was recorded. Low muscle strength was defined as handgrip strength <28 kg for males and <18 kg for females (16).

### Diagnosis of Sarcopenia and Severe Sarcopenia

The AWGS 2019 criteria were used for diagnosis of sarcopenia and severe sarcopenia (16). Briefly, sarcopenia was defined as low muscle mass, plus low physical performance or low muscle strength, while severe sarcopenia was defined as low muscle mass, plus low physical performance, and low muscle strength.

### Other Variables

Information regarding medication that may affect sarcopenia, including insulin, growth or sex hormones, and angiotensin converting enzyme (ACE) inhibitor/angiotensin II receptor blocker (ARB), as well as vitamin D use was obtained. The diagnosis of diabetes mellitus was based on a history of diabetes mellitus or the American Diabetes Association criteria (23). Past history of cerebrovascular disease was defined as prior occurrence of ischemic stroke, transient ischemic attack, intracerebral hemorrhage, and/or subarachnoid hemorrhage. Blood samples were collected from each patient before the start of the first HD session of the week, with standard laboratory parameters including serum albumin, C-reactive protein (CRP), and uric acid levels analyzed using a routine laboratory method at the hospital (24).

### Statistical Analysis

To compare variables between groups, Mann–Whitney's *U*-test (continuous variables) and a chi-squared test (categorical variables) were used. Serum CRP level was logarithmically transformed (log) to follow a normal distribution, before submitting to multivariate logistic regression analysis. Multivariate logistic regression analyses were performed to determine whether XOR inhibitor administration was independently associated with the presence of sarcopenia or severe sarcopenia in addition to that of low muscle mass, low physical performance, or low muscle strength, following adjustments based on various factors, including age, gender, degree of obesity, duration of hemodialysis, cerebrovascular disease, diabetes mellitus, vitamin D, nutritional status, inflammation, and uric acid, each of which are known to be associated with sarcopenia in HD patients (3, 7, 13, 25–28). The consistency of the associations between XOR inhibitor administration and presence of sarcopenia or severe sarcopenia was also examined in relationship to age (≤ />68 years), gender, body mass index (BMI) (≤ />22.6 kg/m^2^), duration of hemodialysis (≤ />6.5 years), cerebrovascular disease (presence/absence), diabetes mellitus (presence/absence), use of oral and/or intravenous vitamin D (presence/absence), serum albumin (≤ />3.6 g/dL), CRP (≤ />0.1 mg/dL), uric acid level (≤ />6.8 mg/dL), diabetic nephropathy (presence/absence), and chronic glomerulonephritis (presence/absence). All statistical analyses were performed using the Statistical Package for the Social Sciences software (PASW Statistics, version 22.0). All reported *p*-values are two-tailed and were considered to be statistically significant at < 0.05.

## Results

### Study Population

Of 305 HD patients 20 years or older who underwent measurements of muscle mass, physical performance, and muscle strength during the study period, those who had been receiving HD therapy for <6 months were excluded (*n* = 3). Furthermore, those with liver cirrhosis, malignancy, infection, or acute illness were also excluded from analysis (*n* = 6). None of the patients were receiving corticosteroid treatment. As a result, 296 HD patients (203 males, 93 females) were retrospectively analyzed. The cause of renal failure was diabetic nephropathy in 87 (29.4%), chronic glomerulonephritis in 80 (27.0%), nephrosclerosis in 25 (8.4%), polycystic kidney disease in 19 (6.4%), graft loss in 16 (5.4%), congenital anomaly in the kidneys or urinary tract in 4 (1.4%), unclassifiable nephritis in 4 (1.4%), kidney or urinary tract tumor in 3 (1.0%), malignant hypertension in 3 (1.0%), pregnancy-induced hypertension in 3 (1.0%), autoimmune nephritis in 1 (0.3%), chronic pyelonephritis in 1 (0.3%), kidney disease-related gout in 1 (0.3%), urinary tract obstruction in 1 (0.3%), other disease in 4 (1.4%), and unknown etiology in 44 (14.9%).

### Clinical Characteristics of Subjects, and Comparisons Between XOR Inhibitor Users and Non-users

Subject characteristics (*n* = 296) are shown in Table 1. The prevalence of sarcopenia and severe sarcopenia in the entire cohort were 42.2% (*n* = 125) and 20.9% (*n* = 62), respectively. Among XOR inhibitor users (*n* = 119; allopurinol: 58, febuxostat, 61), height-adjusted muscle mass and handgrip strength were significantly (*p* < 0.01) higher, and the five-time chair stand test time was significantly lower as compared to the non-users (*n* = 177; Table 1). In addition, the prevalence of low muscle mass (47.1 vs. 65.0%), low physical performance (25.2 vs. 44.1%), and low muscle strength (47.1 vs. 64.4%) was significantly (*p* < 0.01) lower in XOR inhibitor users than in non-users. Finally, the prevalence rates for sarcopenia (33.6 vs. 48.0%) and severe sarcopenia (12.6 vs. 26.6%) were significantly (*p* < 0.05) reduced in the XOR inhibitor users.

**Table 1:** Clinical characteristics of subjects (*n* = 296), and comparisons between XOR inhibitor users and non-users.

	**Total**	**XOR inhibitor users (*n* = 119)**	**XOR inhibitor non-users (*n* = 177)**	**P**
Age, years	68.0 (57.0–74.0)	66.0 (54.0–71.0)	68.0 (61.0–77.0)	0.008
Male, *n*	203 (68.6)	89 (74.8)	114 (66.4)	0.036
BMI, kg/m^2^	22.6 (20.3–25.3)	22.9 (20.7–26.0)	22.5 (19.9–24.8)	0.004
Duration of hemodialysis, years	6.5 (3.0–16.8)	6.0 (2.0–17.0)	7.0 (3.0–16.0)	0.367
Diabetes mellitus, *n*	171 (57.8)	56 (47.1)	115 (65.0)	0.295
Cerebrovascular disease, *n*	42 (14.2)	19 (16.0)	23 (13.0)	0.472
Vitamin D user, *n*	242 (81.8)	97 (81.5)	145 (81.9)	0.279
Insulin user, *n*	33 (11.1)	11 (9.2)	22 (12.4)	0.393
ACE inhibitor/ARB user, *n*	121 (40.9)	52 (43.7)	69 (39.0)	0.419
Albumin, g/dL	3.6 (3.4–3.8)	3.6 (3.4–3.8)	3.6 (3.4–3.8)	0.175
CRP, mg/dL	0.1 (0.1–0.3)	0.1 (0.1–0.4)	0.1 (0.1–0.2)	0.899
Uric acid, mg/dL	6.8 (5.8–7.5)	6.0 (4.9–7.2)	7.1 (6.3–7.7)	<0.001
Height-adjusted muscle mass, kg/m^2^	6.2 (5.6–7.0)	6.7 (5.8–7.5)	6.1 (5.6–6.8)	<0.001
Five-time chair stand test, seconds	10.0 (8.1–13.4)	9.1 (7.7–12.0)	10.9 (8.6–14.9)	0.001
Handgrip strength, kg	24.1 (18.2–29.6)	26.5 (20.2–31.3)	22.5 (16.8–28.0)	0.001
Low muscle mass, *n*	171 (57.8)	56 (47.1)	115 (65.0)	<0.001
Low physical performance, *n*	108 (36.5)	30 (25.2)	78 (44.1)	0.005
Low muscle strength, *n*	170 (57.4)	56 (47.1)	114 (64.4)	0.006
Sarcopenia, *n*	125 (42.2)	40 (33.6)	85 (48.0)	0.003
Severe sarcopenia, *n*	62 (20.9)	15 (12.6)	47 (26.6)	0.013

*Values are expressed as median (interquartile range) or number (%). P-values are shown for comparisons between XOR inhibitor users and non-users. BMI, body mass index; ACE, angiotensin converting enzyme; ARB, angiotensin II receptor blocker; CRP, C-reactive protein; XOR, xanthine oxidoreductase*.

### Associations of XOR Inhibitor Use With Low Muscle Mass, Low Physical Performance, and Low Muscle Strength

To examine whether use of an XOR inhibitor is independently associated with low muscle mass, low physical performance, and/or low muscle strength, multivariate logistic regression analyses were performed (Table 2). XOR inhibitor use was shown to be significantly associated with low muscle mass [odds ratio (OR), 0.384; 95% confidence interval (CI), 0.183–0.806; *p* = 0.011] and low physical performance (OR, 0.286; 95% CI, 0.142–0.578; *p* < 0.001), while a borderline significance with low muscle strength was also noted (OR, 0.500; 95% CI, 0.248–1.006; *p* = 0.052). Additionally, serum uric acid level demonstrated a significant association with low physical performance (OR, 0.710; 95% CI, 0.560–0.899; *p* = 0.005). Furthermore, gender, BMI, and log CRP were significantly associated with low muscle mass, while age, cerebrovascular disease, use of vitamin D, and log CRP were significantly associated with low physical performance, and age, duration of hemodialysis, cerebrovascular disease, and diabetes mellitus were significantly associated with low muscle strength. On the other hand, serum albumin level was not significantly associated with low muscle mass, low physical performance, or low muscle strength (Table 2).

**Table 2:** Multivariate logistic regression analysis of possible factors associated with low muscle mass, low physical performance, and low muscle strength.

	**Low muscle mass**		**Low physical performance**		**Low muscle strength**	
**Variables**	**OR (95% CI)**	***P***	**OR (95% CI)**	***P***	**OR (95% CI)**	***P***
Age	1.015 (0.985-1.045)	0.340	1.084 (1.049–1.120)	<0.001	1.143 (1.101–1.187)	<0.001
Gender (male/female = 1/0)	12.709 (5.695–28.361)	<0.001	0.600 (0.326–1.103)	0.100	1.142 (0.576–2.264)	0.703
BMI	0.608 (0.534–0.693)	<0.001	1.069 (0.993–1.150)	0.076	1.038 (0.958–1.124)	0.360
Duration of hemodialysis	0.996 (0.961–1.033)	0.837	1.026 (0.994–1.059)	0.107	1.089 (1.047–1.134)	<0.001
Cerebrovascular disease (yes/no = 1/0)	2.429 (0.773–7.636)	0.129	3.034 (1.348–6.830)	0.007	4.275 (1.334–13.700)	0.014
Diabetes mellitus (yes/no = 1/0)	1.715 (0.825–3.564)	0.148	1.062 (0.575–1.958)	0.848	2.536 (1.266–5.083)	0.009
Vitamin D (yes/no = 1/0)	1.902 (0.848–4.267)	0.119	0.443 (0.213–0.922)	0.030	0.518 (0.233–1.151)	0.107
Albumin	0.460 (0.145–1.462)	0.188	0.790 (0.285–2.187)	0.649	0.396 (0.127–1.235)	0.111
Log CRP	6.429 (1.938–21.333)	0.002	3.001 (1.238–7.274)	0.015	1.250 (0.455–3.434)	0.665
Uric acid	0.925 (0.727–1.176)	0.524	0.710 (0.560–0.899)	0.005	0.816 (0.636–1.047)	0.110
XOR inhibitor (yes/no = 1/0)	0.384 (0.183–0.806)	0.011	0.286 (0.142–0.578)	<0.001	0.500 (0.248–1.006)	0.052

*BMI, body mass index; CRP, C-reactive protein; XOR, xanthine oxidoreductase; OR, odds ratio; CI, confidence interval*.

### Associations of XOR Inhibitor Use With Sarcopenia and Severe Sarcopenia

**Figure 1:**
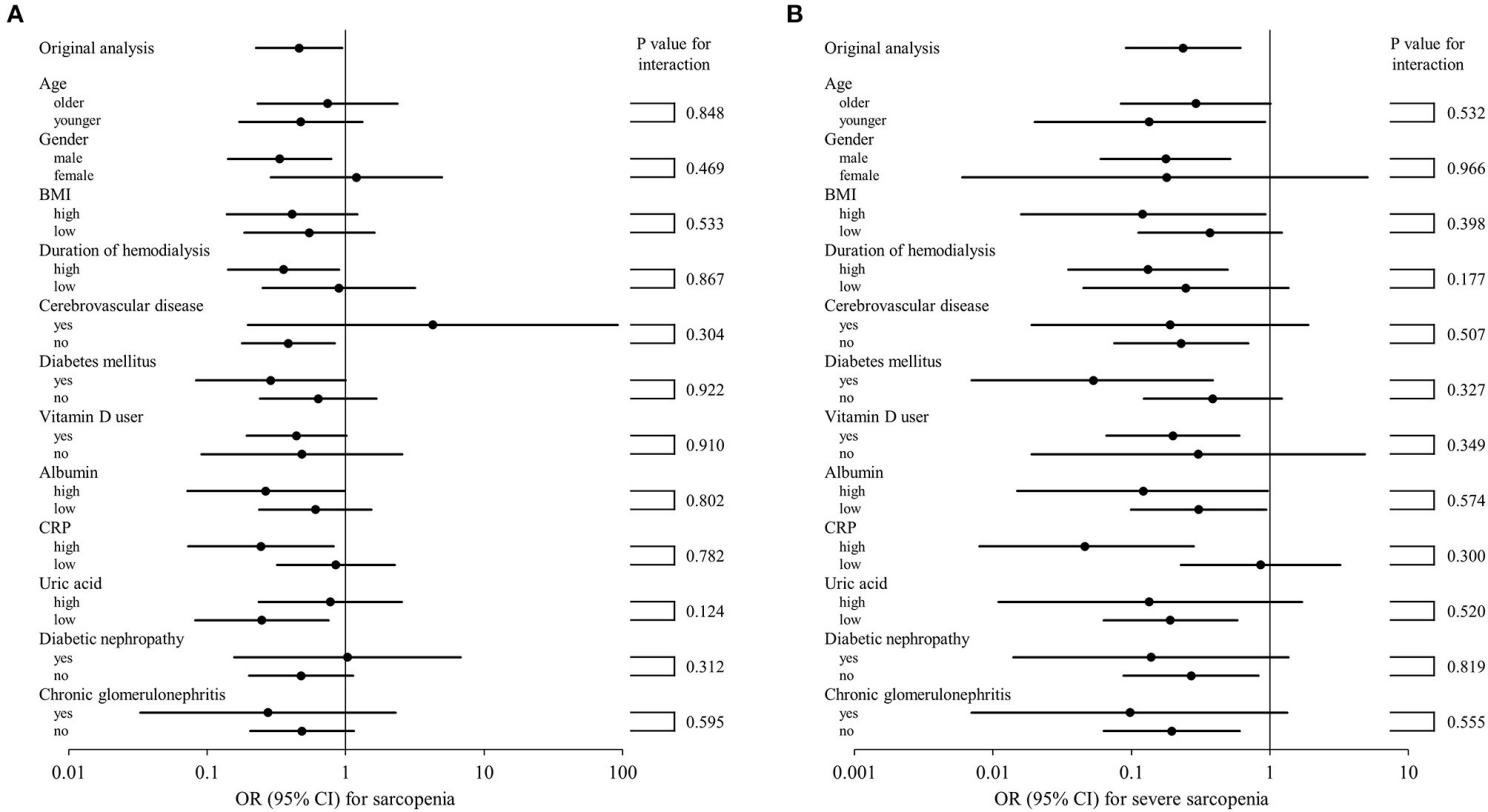
Subgroup analysis of association of XOR inhibitor use with sarcopenia **(A)** and severe sarcopenia **(B)**. Forrest plots showing OR (95% CI) in squares and lines for each category are presented. The 95% CI values shown were calculated within each category and *p*-values for subgroup analyses represent tests for interaction. The higher and lower groups were defined as greater and less than or equal to the median, respectively. BMI, body mass index; CRP, C-reactive protein; XOR, xanthine oxidoreductase; OR, odds ratio; CI, confidence interval.

To further examine whether XOR inhibitor use is independently associated with sarcopenia or severe sarcopenia, multivariate logistic regression analyses were again performed (Table 3). These results showed that use of an XOR inhibitor was significantly associated with sarcopenia (OR, 0.462; 95% CI, 0.226–0.947; *p* = 0.035) and severe sarcopenia (OR, 0.236; 95% CI, 0.091–0.614; *p* = 0.003). Notably, there was no remarkable inconsistency observed among the results for the series of subgroups (Figures 1A,B). Age, gender, BMI, and history of cerebrovascular diseases were significantly associated with both sarcopenia and severe sarcopenia, while serum uric acid as well as CRP level were significantly associated with severe sarcopenia (OR, 0.715; 95% CI, 0.524–0.977; *p* = 0.035; Table 3). In contrast, serum albumin level, duration of hemodialysis, diabetes mellitus, and vitamin D use were not significantly associated with sarcopenia or severe sarcopenia (Table 3). When presence of XOR inhibitor was replaced by type and presence of XOR inhibitor, use of allopurinol and febuxostat (ref. absence of XOR inhibitor) was similarly associated with sarcopenia (OR 0.435, 95% CI 0.185–1.027, *p* = 0.058; OR 0.509, 95% CI 0.182–1.425, *p* = 0.198, respectively) and severe sarcopenia (OR 0.298, 95% CI 0.099–0.892, *p* = 0.030; OR 0.163, 95% CI 0.042–0.634, *p* = 0.009, respectively), suggesting that differences among types of XOR inhibitors have little effect on sarcopenia/severe sarcopenia. When use of insulin or ACE inhibitors/ARBs was added to the multivariate logistic regression model, use of XOR inhibitors remained significantly associated with sarcopenia (OR 0.467, 95% CI 0.228–0.957, *p* = 0.038; OR 0.463, 95% CI 0.226–0.948, *p* = 0.035, respectively) and severe sarcopenia (OR 0.238, 95% CI 0.091–0.620, *p* = 0.003; OR, 0.236, 95% CI 0.091–0.614, *p* = 0.003, respectively).

**Table 3:** Multivariate logistic regression analysis of possible factors associated with sarcopenia and severe sarcopenia.

	**Sarcopenia**		**Severe sarcopenia**	
**Variables**	**OR (95% CI)**	***P***	**OR (95% CI)**	***P***
Age	1.090 (1.054–1.127)	<0.001	1.086 (1.041–1.133)	<0.001
Gender (male/female = 1/0)	8.746 (4.085–18.724)	<0.001	4.178 (1.768–9.874)	0.001
BMI	0.799 (0.726–0.880)	<0.001	0.773 (0.684–0.874)	<0.001
Duration of hemodialysis	1.029 (0.995–1.064)	0.101	1.013 (0.975–1.052)	0.510
Cerebrovascular disease (yes/no = 1/0)	3.649 (1.370–9.721)	0.010	2.703 (1.106–6.608)	0.029
Diabetes mellitus (yes/no = 1/0)	1.769 (0.911–3.437)	0.092	1.063 (0.493–2.289)	0.876
Vitamin D (yes/no = 1/0)	0.713 (0.332–1.533)	0.387	0.431 (0.178–1.039)	0.061
Albumin	0.421 (0.139–1.273)	0.126	0.665 (0.187–2.372)	0.530
Log CRP	1.421 (0.529–3.823)	0.486	4.254 (1.501–12.054)	0.006
Uric acid	0.836 (0.653–1.071)	0.157	0.715 (0.524–0.977)	0.035
XOR inhibitor (yes/no = 1/0)	0.462 (0.226–0.947)	0.035	0.236 (0.091–0.614)	0.003

*BMI, body mass index; CRP, C-reactive protein; XOR, xanthine oxidoreductase; OR, odds ratio; CI, confidence interval*.

## Discussion

In this first known study to investigate the association of XOR inhibitor treatment with sarcopenia in HD patients, the following primary findings were obtained. First, the rates of prevalence of sarcopenia and severe sarcopenia, determined based on the AWGS 2019 criteria, were 42.2 and 20.9%, respectively (Table 1). Furthermore, XOR inhibitor use was shown to be significantly associated with reduced risk of low muscle mass and low physical performance, while a borderline significance with low muscle strength was also found (Table 2). Finally, XOR inhibitor use was significantly associated with reduced risk of both sarcopenia and severe sarcopenia (Table 3). Together, the present results suggest that treatment with an XOR inhibitor exerts protective effects to reduce the risk of sarcopenia and severe sarcopenia, which are frequently observed in HD patients.

A diagnostic algorithm for sarcopenia has been proposed by several different western groups, including the Foundation for the National Institutes of Health Sarcopenia Project (29), European Working Group on Sarcopenia in Older People (30, 31), and International working group on sarcopenia (32). However, because of anthropometric as well as cultural and/or lifestyle-related differences in Asia as compared with Western populations, the AWGS proposed a diagnostic algorithm for sarcopenia based on Asian data in 2014 (33), which was updated in 2019 (16). Previous studies including ours have reported the prevalence of sarcopenia in HD patients based on the AWGS 2014 criteria as ranging from 33.7 to 40% (2, 3), though no studies reporting that prevalence in HD patients based on the AWGS 2019 criteria have been presented prior to the present report (Table 1).

Previous clinical studies that examined Tour de France cyclists and professional soccer players have found that administration of an XOR inhibitor protects against skeletal muscle damage caused by exhaustive exercise (34, 35). Furthermore, other studies have noted that XOR inhibitor administration protects against skeletal muscle atrophy caused by immobilization following an ankle sprain in male subjects (36) and improved functional outcomes after rehabilitation in older patients (37), suggesting that XOR inhibitor administration contributes to protect against sarcopenia. However, to the best of our knowledge, no investigation of the association of XOR inhibitor treatment with sarcopenia has been presented. The present results are the first to demonstrate associations of XOR inhibitor use with reduced levels of sarcopenia and severe sarcopenia (Table 3), as well as its protection against low levels of muscle mass, physical performance, and muscle strength (Table 2).

Although it remains unclear why XOR inhibitor use has been found to be associated with those factors, decreased ROS production could be a potential reason. XOR is involved in production of ROS and uric acid, and expressed in the vascular endothelium of skeletal muscle (38), as well as in the liver and intestines (39, 40). Previous studies have shown that administration of an XOR inhibitor protected against skeletal muscle atrophy caused by unloading in mice and rats by inhibiting activation of the ubiquitin-proteasome pathway via decreased ROS production in skeletal muscle (36, 41). In addition, XOR inhibitor administration was reported to increase maximal isometric force in aged mice by decreasing ROS production in skeletal muscle (42). Therefore, treatment with an XOR inhibitor may contribute to preservation of skeletal muscle mass and improvement of skeletal muscle function by reducing ROS production in skeletal muscle. On the other hand, increased uric acid level was found to be significantly associated with a reduced risk of low physical performance (Table 2) and severe sarcopenia (Table 3). Uric acid has been shown to gain antioxidant properties by scavenging ROS in a hydrophilic condition, such as by circulating in blood (43), and when present in the circulation is thought to have a protective effect against sarcopenia (44–46). Since renal excretion of uric acid is reduced in HD patients, administration of an XOR inhibitor may exert a protective effect against sarcopenia by increasing the level of uric acid level relative to XOR activity in circulating blood.

Another possibility that can potentially explain the protective effects of XOR inhibitor treatment is its effect to conserve the intracellular level of ATP, thus providing energy necessary for muscle contractions (47). In skeletal muscle, ATP production has shown to be decreased under a uremic condition (48) and ATP levels have been shown to be significantly lower in uremic patients as compared to control subjects (15, 49). Of importance, administration of an XOR inhibitor enhances the intracellular ATP level by increasing the purine salvage pathway through use of hypoxanthine and decreasing energy expenditure by reducing *de novo* purine synthesis (9–12). Taken together, these findings indicate that XOR inhibitor treatment might exert protective effects on sarcopenia in HD patients through preservation of skeletal muscle mass and improvement of skeletal muscle function by ATP enhancement in skeletal muscle.

Previous studies have found associations of age, gender, degree of obesity, duration of hemodialysis, cerebrovascular disease, diabetes mellitus, vitamin D use, inflammation, and uric acid with sarcopenia or its components in HD patients (3, 7, 13, 25–27). In the present investigation as well, age, gender, BMI, duration of hemodialysis, history of cerebrovascular disease, presence of diabetes mellitus, use of oral and/or intravenous vitamin D, CRP, and uric acid level were shown to be significantly associated with some or all of the factors low muscle mass, low physical performance, low muscle strength, sarcopenia, and severe sarcopenia. On the other hand, while nutritional status has also been reported to be associated with sarcopenia in HD patients (28), serum albumin level showed no significant association with low muscle mass, low physical performance, low muscle strength, sarcopenia, or severe sarcopenia in our study (Tables 2, 3). Although serum albumin level is considered to be an indicator of nutritional status, that level may not adequately reflect nutritional status in HD patients, since it did not show a significant association with sarcopenia or related components. In addition, nutritional intake has been reported to be associated with sarcopenia in older subjects (50), though unfortunately information regarding nutritional intake in the present subjects was not obtained. In future investigations of the relationship between XOR inhibitors and sarcopenia, it will be necessary to include accurate information regarding nutritional intake and status in the analysis.

This study has several limitations. First is the cross-sectional design, thus even though relationships were explored in predictive terms, the results cannot be interpreted to show causal relationships. Second, while physical activity has been shown to influence XOR activity (51–53), no survey of physical activity was conducted in the present study. Furthermore, we were unable to fully investigate the association of dosage, duration, or indication of XOR inhibitor administration with sarcopenia. Third, because of the methods used, ROS, oxidative stress, and ATP levels in skeletal muscle and blood were not determined. Fourth, since muscle mass, physical performance, and muscle strength were examined as part of routine clinical care, patients unable to perform related tests due to reduced activities of daily living were not included in the present study. Fifth, there were few cases of severe sarcopenia, thus the 10 events per variable rule (54) could not be used when performing multivariate logistic regression for severe sarcopenia. A large-scale prospective interventional study that includes measurements of ROS, oxidative stress, and ATP levels in skeletal muscle and blood, as well as physical activity, and also includes analysis of dosage, duration, and XOR inhibitor indication in HD patients, including those with markedly reduced activities of daily living, is needed to clarify the role of XOR inhibitor treatment for prevention and treatment of sarcopenia in HD patients. Finally, the present study population consisted of nearly exclusively Japanese patients with HD, thus it is unclear whether the findings can generalized for other ethnic groups or non-HD subjects.

In conclusion, the present results showed that XOR inhibitor use in HD patients is significantly associated with reduced risk of sarcopenia and severe sarcopenia, as well as low muscle mass and low physical performance, based on the AWGS 2019 criteria.

## Data Availability Statement

The original contributions presented in the study are included in the article/supplementary material, further inquiries can be directed to Masafumi Kurajoh, m1155129@med.osaka-cu.ac.jp.

## Ethics Statement

The studies involving human participants were reviewed and approved by the Inoue Hospital Ethics Committee. The ethics committee waived the requirement of written informed consent for participation.

## Author Contributions

MK contributed to study design, interpretation, and writing of the manuscript. KM, MM, SM, and AK contributed to study design and interpretation. YT contributed to study design, data analysis, and interpretation. ME reviewed the manuscript. All authors have read and approved the final version of the manuscript.

## Conflict of Interest

The authors declare that the research was conducted in the absence of any commercial or financial relationships that could be construed as a potential conflict of interest.

## Publisher's Note

All claims expressed in this article are solely those of the authors and do not necessarily represent those of their affiliated organizations, or those of the publisher, the editors and the reviewers. Any product that may be evaluated in this article, or claim that may be made by its manufacturer, is not guaranteed or endorsed by the publisher.
